# Salt Effects on the Phase Behavior and Cocrystallization Kinetics of POCB–Water Mixtures

**DOI:** 10.1021/acs.langmuir.3c02428

**Published:** 2024-02-02

**Authors:** Michael Gresh-Sill, Sudesna Banerjee, Tara Y. Meyer, Sachin S. Velankar

**Affiliations:** † Department of Chemical Engineering, University of Pittsburgh, 940 Benedum Hall, Pittsburgh 15261, Pennsylvania, USA; ‡ Department of Chemistry, University of Pittsburgh, Chevron Science Center, 219 Parkman Ave, Pittsburgh 15260, Pennsylvania, USA; § Department of Mechanical Engineering, University of Pittsburgh, 636 Benedum Hall, Pittsburgh 15261, Pennsylvania, USA

## Abstract

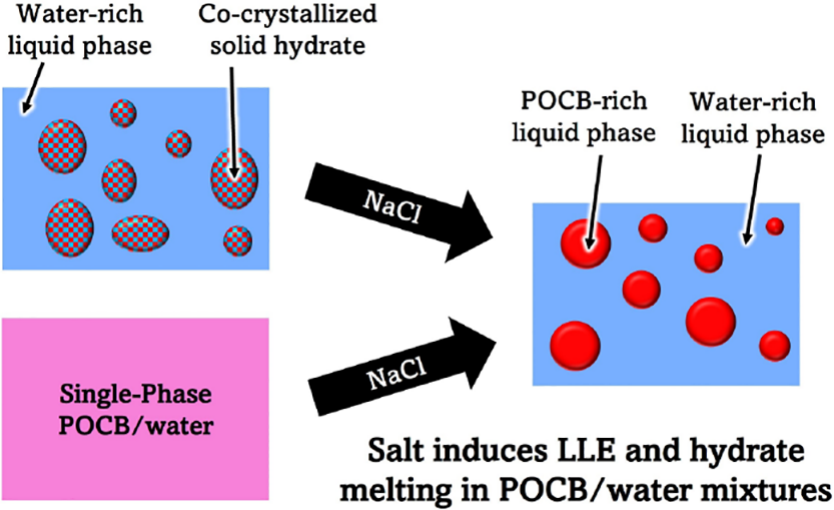

Mixtures of water with polyoxacyclobutane (POCB) have a unique phase diagram which combines liquid–liquid equilibrium (LLE) at high temperatures and cocrystallization of a POCB-hydrate at low temperatures. Such cocrystal hydrate formation is extremely rare among polymers. We report on the effects of adding NaCl salt on the phase behavior of POCB–water mixtures and the kinetics of hydrate crystallization from such mixtures. Salt loadings of less than 0.1 wt % were found to greatly expand the LLE region. Salt loadings of ∼10 wt % were found to significantly decrease the melting temperature of the hydrate below its ∼37 °C value under salt-free conditions. The hydrate was found to be remarkably tolerant of salt and persists at room temperature even when equilibrated with salt-saturated water. Salt was found to slow down hydrate crystallization, and the degree of slowing was greater than that expected from the salt-induced decrease in undercooling due to melting point depression.

## Introduction

Polyoxacyclobutane (POCB) is a polymer in the polyoxyalkylene series with the structure– [(CH_2_)_3_O-]_*n*_. Mixtures of water with POCB show unusual phase behavior. At room temperature and atmospheric pressure, POCB has a remarkable ability to cocrystallize with water to form a hydrate.^1^ However, above the melting temperature of the hydrate, one obtains a mixture in liquid–liquid equilibrium (LLE) between a POCB-rich and a water-rich phase. Such demixing indicates that—despite its apparent affinity for water in the crystalline hydrate state—POCB is hydrophobic.^2^ Indeed, all other polyoxyalkylenes (except poly(ethylene oxide)) are also hydrophobic with low solubility in water. This combination of properties makes POCB unique: to our knowledge, it is one of only two^3^ (possibly three^4^) polymers that can form a crystalline hydrate, and the only one that also phase separates from water under molten conditions. In fact, to our knowledge, no other polymer is able to cocrystallize with a small molecule with which it is immiscible in the liquid state.^5^

**Figure 1:**
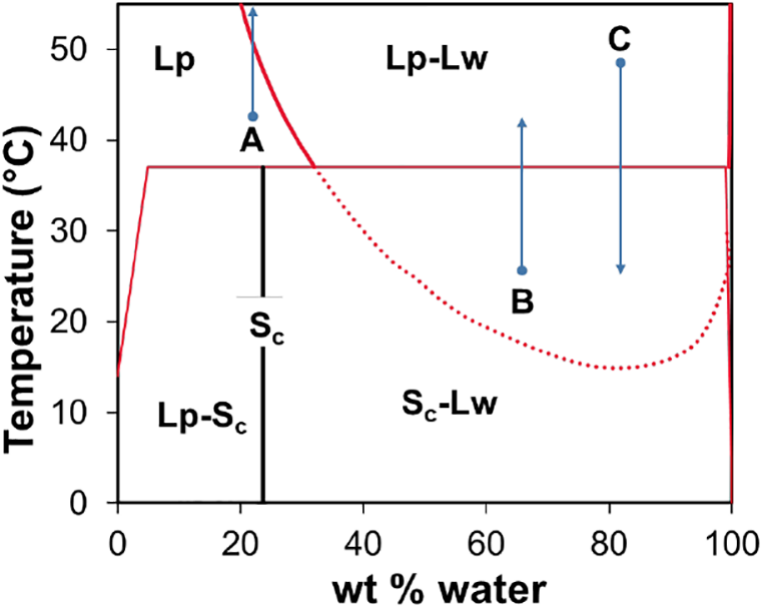
Phase diagram for POCB 650 and water without salt.^2^ The solid black line labeled Sc indicates the composition of the solid hydrate. The solid red curve indicates liquid–liquid coexistence between the polymer-rich (Lp) and water-rich (Lw) phases, whereas the horizontal red line is the melting point of the hydrate. The dotted red line indicates a metastable portion of the liquid–liquid coexistence curve. The blue arrows indicate the trajectories used for the measurements of interest in this study. (A) Heating a single-phase mixture to locate the cloud point. (B) Heating a hydrate–liquid mixture to measure the melting point. (C) Cooling a two-phase mixture in LLE to measure the crystallization kinetics. This diagram is not strictly correct, as discussed in the Results and Discussion section.

Previously, we conducted studies of the phase behavior and hydrate crystallization kinetics of POCB–water mixtures.^6,7^ Briefly, at a molecular weight of 650 g/mol, the phase diagram is a union of hydrate melting behavior and LLE behavior^2^ (Figure 1, discussed in detail in the Results and Discussion section). Accordingly, mixtures with low water content can be cooled from a homogeneous liquid state to form a hydrate, and this crystallization process resembles homopolymer crystallization.^6^ In contrast, mixtures with a high water content are in LLE prior to crystallization. Thus, hydrate formation involves interfacial mass transport and is, therefore, sensitive to mixing conditions.^7^

POCB-hydrate formation has many potential applications including improving the nonvolatile memory storage capabilities of carbon nanotube devices,^8^ drug delivery or other biomedical applications,^9,10^ water purification,^11^ materials with high proton and ion conductivity for membrane applications,^12,13^ and stimuli-responsive materials.^14^ Ionic solutes are expected to be present in virtually all of these applications. The effect of ionic solutes on the phase behavior and hydrate crystallization kinetics of POCB/water mixtures is unknown and the subject of this paper. Salt is known to affect the solubility of polymers through the Hofmeister effect.^15^ Thus, we anticipate that salt promotes the immiscibility of POCB and water, and indeed this was noted previously^16^ as discussed further below. Additionally, any solute can reduce the melting point of a crystal. Thus, salt is expected to affect the phase boundaries of both the LLE and solid–liquid equilibrium in the POCB–water mixtures. These changes in the phase behavior are also likely to affect the crystallization kinetics. For example, melting point depression of the hydrate would reduce the undercooling (at any specified crystallization temperature), and hence, the addition of salt would also slow crystallization. The goal of this paper is to measure these effects quantitatively.

We are aware of only one previous publication of salt effects on POCB/water mixtures.^16^ Lee et al. showed that salt-free POCB/water mixtures that were transparent, homogeneous mixtures at room temperature reached a cloud point (i.e., demixed) upon heating, and further that the cloud point temperature reduced upon the addition of NaCl. This research was conducted at a POCB molecular weight of 250 g/mol^16^ and did not mention hydrate formation at all. Indeed, our experiments with a similarly low molecular weight show that the hydrate does not form readily. The research in this paper will focus on the effects of NaCl as the salt but will use a MW of 650 g/mol of POCB at which hydrate crystallization and LLE are coupled with each other. It is this coupling that makes this system unique, and there have been no previous studies of salt effects on any similar polymer–water mixture.

The salt-induced demixing seen in POCB/water mixtures previously^16^ and in this paper is common across a variety of water-soluble polymers, wherein the solubility of the polymer in water reduces with increasing salt content. These include polymers of the polyoxyalkene series, such as short chain polyoxymethylene (POM), poly(ethylene oxide) (PEO), poly(propylene oxide) (PPO), and POCB, as well as other polymers such as polyvinylpyrrolidone (PVP), poly(*N*-isopropylacrylamide) (PNIPAM), or poly(2-oxazoline) (POX).^17−24^ Similar salt effects are also seen in other aqueous nonionic polymer mixtures such as dextran–polyvinylpyrrolidone, dextran–poly(vinyl alcohol), dextran–ficoll, and dextran–PEG.^25^ These aqueous systems have been known since the late 1800s and have been studied for their use in biological separations since Albertsson’s group pioneered the practice in the 1950s.^15,26^

In contrast to the well-established literature on salt effects on LLE in water–polymer mixtures, we are unaware of any studies on the effects of salt on crystallization of polymer hydrates. There are, however, limited studies of salt effects on homopolymer crystallization in the absence of water. Salt can significantly slow down the kinetics of crystallization,^27−29^ which is at least partially attributed to the reduction in the equilibrium melting temperature of the homopolymer, and thereby a reduction of undercooling. Any reduction beyond the decrease in undercooling may be attributable to more complex effects such as change in surface energy at the crystal–amorphous interface or transport limitations to crystallization. Incidentally, outside the polymer science literature, salt effects on gas hydrates or other small-molecule clathrate hydrates are well studied. Salt is known to reduce the melting temperature of the hydrate, as well as slow down crystallization.^30^ However, these clathrate hydrates are very different: they have a water-cage structure (vs the intercalated structure of POCB-hydrate); the water content of gas hydrates is far higher than in POCB-hydrate; and the growth mechanism and habit of gas hydrates are entirely distinct from those for POCB hydrate.

## Results and Discussion

The results section is organized into two parts. The Salt Effects on Phase Behavior subsection describes the effects of salt on the phase diagram, both on how salt affects LLE (as judged by cloud point measurements), and the melting temperature of the hydrate. In both of these cases, we report changes in phase behavior at a fixed polymer fraction while varying the salt content and fixed salt content while varying the polymer fraction. The Crystallization Kinetics subsection describes the effect of salt on the kinetics of hydrate crystallization. Details of the experimental methods and data analysis are given at the end of the paper.

### Salt Effects on Phase Behavior

#### Liquid–Liquid Equilibria

As shown in Figure 1, at the molecular weight of 650 g/mol, in the absence of salt, the LLE behavior had the following characteristics. Above 37 °C (the melting temperature of the hydrate), the mixtures are in LLE between water-rich and POCB-rich phases. The miscibility gap (i.e., the difference between the compositions of the water-rich and the POCB-rich phases) widens at higher temperatures, suggesting that the LLE curve has a lower-consolute solution temperature (LCST). Below 37 °C, while mixtures may remain in LLE for short periods, the hydrate forms eventually. This portion of the LLE curve is metastable, and we were not able to quantify it fully.

It must be noted that Figure 1 is schematic, and hence, not strictly correct. First, at very low polymer (or very low water) content, there must be a small region of melting point depression of ice (or of pure solid POCB). These regions could not be explored experimentally since the corresponding polymer or water contents are too low. Second, the hydrate melting points did not show measurable changes with composition, and hence, the hydrate melting line is shown to be horizontal. While this is correct for the three-phase (Lp–Lc–Sc) coexistence region, it may not be true for the Lp–Sc coexistence since the hydrate melting point must be depressed for compositions near the crystal composition (23.6 wt % water). Evidently, this melting point depression is too small to be measured in our experiment. Third, at low water content, crystallization takes a long time and thermodynamic equilibrium may not be maintained. Nevertheless, the phase diagram in Figure 1 is consistent with our experiments and suitable for guiding this paper.

**Figure 2:**
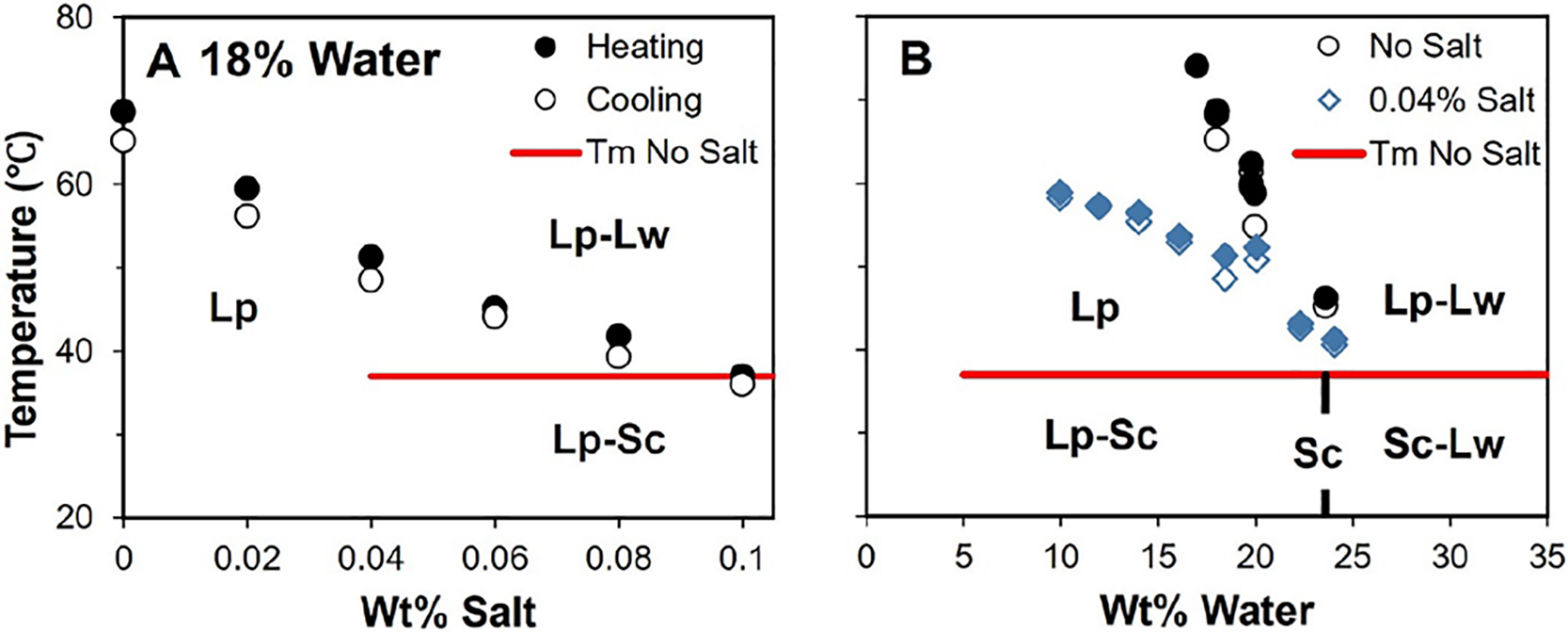
Cloud points for POCB/water/salt mixtures. (A) Cloud points for samples with 18% water vs salt content. Below these cloud points, the mixtures are single-phase liquids, whereas two liquid phases (Lp and Lw) exist above the cloud points. (B) Cloud points for samples without salt and with 0.04% salt at various water contents. Samples are single-phase below the black circles for the salt-free samples and below the blue diamonds at 0.04% salt. The vertical black line labeled Sc indicates the composition of the POCB–water hydrate. In both graphs, the open symbols represent cloud points measured during cooling, and the closed symbols represent cloud points measured during heating. In both graphs, the solid red line is the melting point of the hydrate, which is nearly independent of salt content (see text) and of water content.^2^ In part B, the red line does not extend below 5% water because slow crystallization makes it difficult to study hydrate melting. A single pair of heating–cooling points is shown in each graph. Yet, the reproducibility of two independently prepared samples is better than the difference between the heating–cooling pair shown.

Turning now to the effects of salt, a first set of experiments was conducted at fixed water content (18 wt %) to gauge the amount of salt needed to induce a noticeable depression in the cloud point. Figure 2A shows the cloud point for samples with 0.0 to 0.1 wt % salt. The cloud point is found to reduce from around 67 °C for the salt-free mixture to around 36 °C at 0.1% salt. Above 0.1% salt, the cloud points are below the melting temperature of the hydrate; therefore, mixtures in LLE must eventually crystallize. While nonequilibrium cloud points can be measured, in the present case, hydrate crystallization was too rapid to allow such quantification.

The water-rich branch of the cloud point curve was not quantified in this research. Previously we noted that in the absence of salt, the addition of even 1% POCB (MW 650 g/mol) to pure water brings the mixture into the LLE. Thus, the boundary of the LLE region extends to nearly pure water, making it difficult to quantify precisely. This remains true with the addition of salt.

The magnitude of cloud point depression observed here is far larger than in Lee et al.^16^ who found that a similar 30 °C drop required a salt loading of 5.0 wt % vs less than 0.1 wt % in Figure 2A. This difference may be attributable to the differences in MW between our samples (650 g/mol) vs Lee (250 g/mol). Indeed, in solutions of other polymers such as PEO, PVP, and PNIPAM,^18,21−23^ the magnitude of cloud point depression with added salt is known to increase with MW.

Based on the results of Figure 2A, a second set of experiments was then conducted at a fixed salt content of 0.04 wt %, varying the water content from 10 to 20 wt %. Figure 2B shows that the magnitude of cloud point reduction is more severe as the water content reduces. This latter observation may be rationalized as follows.

The common explanation for the salt effect on cloud point depression is a result of polymer–ion interactions and competition for hydration.^18,19,26,31−33^ Qualitatively, the ions avoid association with the polymer and instead prefer to associate with water. This reduces the level of hydration of the polymer, eventually inducing the formation of a separate, polymer-rich liquid phase. Indeed, this effect is not limited to polymers; homogeneous mixtures of water with organic solvents can be induced to phase separate upon adding salt.^26^ As the water content reduces while the salt loading is held fixed, the amount of water available for hydrating the polymer reduces steeply, thus, making LLE more favorable. Therefore, samples with lower water content show a larger magnitude of cloud point depression in Figure 2B.

Finally, a complete description of LLE also requires examination of the partitioning of the salt into the two coexisting phases. We presume that salt would preferentially partition into the water-rich phase, with very little being present in the POCB-rich phase. This was verified in a single experiment (see ESI), which establishes that the POCB-rich phase can dissolve at most 0.01% salt at 40 °C, even when the aqueous phase is saturated saltwater. When the aqueous phase is much more dilute, e.g., as in Figure 2, we presume that the POCB-rich phase has even lower amounts of salt.

#### Melting Point

Preliminary experiments showed that the mixtures from Figure 2A (with up to 0.1% salt) induced negligible melting point depression; several percent salt was needed to reduce the hydrate melting point measurably. Accordingly, separate samples were made at higher salt contents.

**Figure 3:**
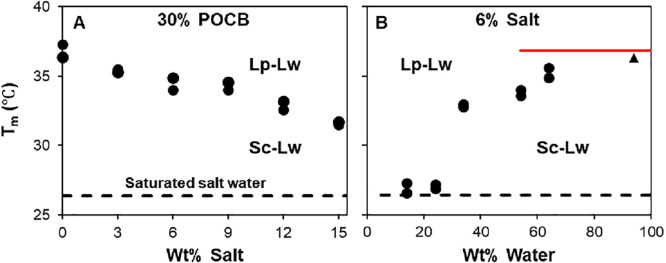
(A) The hydrate melting temperature obtained from optical microscopy for varying salt contents and fixed POCB loading of 30 wt %. (B) Melting temperature for varying water contents with a fixed salt loading of 6 wt %. The solid red line indicates the melting point for hydrate crystals with no salt present. The dashed line indicates the melting temperature of hydrate crystals in equilibrium with saturated salt water. The black triangle denotes a dilute POCB sample in 6% saltwater.

Similar to the previous section, initial experiments were conducted at a fixed water loading to identify the salt content needed to see a measurable decrease in the melting temperature. Similar to previous experiments,^2^ we first cooled the mixtures (which are initially in LLE) in vials to form hydrate, and then gradually heated them under microscope to observe the highest temperature at which hydrate crystals still persist. Figure S1 shows exemplary images during the melting process. These experiments were conducted at 30% POCB, i.e., a much higher water content than in Figure 2 because at 30% POCB, mixtures develop a waxy solid-like consistency during crystallization, making it easy to transfer from the vial to the microscope stage. In contrast, samples with much higher or lower water content undergo large-scale phase separation and sedimentation; and hence, the composition of the sample transferred to the microscope deviates from the original sample. Samples of fixed POCB content (30 wt %) were prepared with salt loadings varying from 0.0 to 15.0 wt %. Their melting points, *T*_m_ (Figure 3A) were found to reduce by several degrees over the range of salt content examined.

It is interesting to consider the state of the mixtures in Figure 3A prior to melting. We define *m*_s_, *m*_p_, and *m*_w_ as the weight fractions of the salt, polymer, and water in the overall mixture and take a unit mass of the mixture as basis. If χ is the fraction of the POCB that crystallizes to form hydrate, then the mass of water consumed in hydrate formation is 0.31*χm*_p_ per unit mass of mixture (since the hydrate has a POCB:water ratio of 1:0.31). The salt fraction in the water-rich (Lw) phase is now
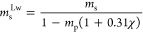
1The above assumes that the POCB-rich phase is nearly pure, the water-rich phase has negligible dissolved POCB, and all the salt partitions into the water-rich phase. For the highest salt loading in Figure 3A, *m*_s_*=* 0.15 and *m*_p_ = 0.3. If we assume full crystallization, χ = 1.0, eq 1 gives . This last number is close to the weight fraction of salt in salt-saturated water (roughly 0.27), i.e., as per this calculation this fully crystallized mixture would have an aqueous phase that is nearly saturated with salt. The above estimate of the salt fraction of 0.247 is an upper bound, since it assumes full crystallization of POCB, while polymers never crystallize to their full extent. In fact, we noted previously,^6,7^ that in the absence of salt, the volume change of crystallization was approximately half of that expected for full hydrate crystallization. Thus, if we take χ = 0.5, eq 1 gives , which is still relatively close to full saturation. This calculation raises an interesting question: can the hydrate coexists with saturated water, and if so, what is the melting temperature of the hydrate in equilibrium with salt-saturated water?

To address this, a separate experiment was conducted. A small amount of POCB was added to pure water to precipitate hydrate crystals. A small quantity of the crystals was transferred to a vial of salt water with excess salt (i.e., the aqueous phase is salt-saturated water), which was then heated gradually until the hydrate crystals melted. The corresponding temperature (roughly 26 °C), indicated by the dashed line in Figure 3, serves as a lower bound for the melting point in Figure 3, and corresponds to four-phase equilibrium between hydrate, salt crystals, a POCB-rich liquid phase, and saturated salt water (with very little POCB). It is noteworthy that this temperature exceeds room temperature, i.e., the hydrate is sufficiently salt-tolerant that does not melt even when equilibrated with salt-saturated water at room temperature.

Finally, Figure 3B shows the dependence of melting point on composition at a fixed salt content of 6 wt %. The point marked with a black triangle represents an experiment in which a small amount of hydrate crystal particles was placed in 6% saltwater and then melted. The corresponding hydrate melting point of *T*_m_ = 36.3 °C shows no significant melting point depression as compared to the melting of a hydrate in equilibrium with pure water. With decreasing overall water content, the melting point reduces precipitously once the overall water loading in the mixture decreases below 40 wt %, and levels off at about 27 °C, close to the value for the hydrate in equilibrium with salt-saturated water. This may be rationalized from eq 1, once again taking χ = 0.5 for the following example calculations. The overall water loading of 34% in Figure 3B corresponds to *m*_s_ = 0.06, *m*_p_ = 0.6, and *m*_w_ = 0.34. For this composition, eq 1 gives , thus, at 34% overall water, with crystallization of half of the POCB, the aqueous phase is still well below the saturation point. However, at an overall water loading of 24%, the mixture composition is *m*_s_*=* 0.06, *m*_p_ = 0.7, and *m*_w_ = 0.24. For this composition, eq 1 gives , corresponding to a supersaturated salt solution. Therefore, the steep drop in *T*_m_ below 40% water and the plateau around *T*_m_ = 27 °C at low water loading are consistent with the aqueous phase first approaching saturation and then becoming supersaturated. Indeed, salt crystals were observed in samples with 70% and 80 wt % POCB prior to melting. Since, the water-rich phase is salt-saturated, it is unsurprising that *T*_m_ shows a plateau at a value similar to that of melting hydrate crystals placed into a vial of salt-saturated water.

### Crystallization Kinetics

The study of phase behavior from the previous section guides our experiments on hydrate crystallization kinetics in two important ways. First, Figure 3 shows that the salt lowers the equilibrium melting temperature. Thus, at any specified crystallization temperature, *T*_c_, increasing salt loading reduces the undercooling (*T*_m_ – *T*_c_) and is expected to slow down crystallization kinetics. The second is that in mixtures containing over 0.1% salt, above *T*_m_, LLE prevails at almost all POCB:water ratios (Figure 2). Thus, prior to crystallization, the mixture is almost always in LLE, and hence, three phases (two liquid, and the solid hydrate) are present during crystallization.^6,7^ Moreover, in general, neither of the two liquid phases coexisting during crystallization has the same composition as the hydrate. Instead, the water-rich phase is dilute in POCB, whereas the polymer-rich phase is dilute in water; the latter, especially, may change with salt content and crystallization temperature. Therefore, in general, hydrate crystallization may involve interfacial mass transport and hence is expected to depend on the mixing conditions. Accordingly, differential scanning calorimetry, the common method for studying the crystallization kinetics of polymers, cannot be used. Instead, we examined the hydrate crystallization kinetics by dilatometry,^7^ stirring the dilatometer flasks continuously with magnetic stir bars to avoid density-based separation. This is possible only at relatively low viscosity, necessitating a relatively low POCB content. Accordingly, our kinetic study was restricted to POCB–water mixtures with a low POCB content of 20 wt %. The kinetics data on salt-free samples were discussed comprehensively in a separate study by Banerjee et al.^7^

Mixtures with 20 wt % POCB, salt loading ranging from 0.0 to 7 wt %, and the remainder water were prepared in a dilatometer with a magnetic stirrer. For each experiment, samples were held above their melting point in a hot bath at 48 °C and then transferred to a second bath held at a fixed crystallization temperature *T*_c_. Volume changes were measured by quantitative video analysis over the course of 3–6 h. 2–4 samples were prepared at each individual composition, and each sample was crystallized isothermally at various values of *T*_c_.

**Figure 4:**
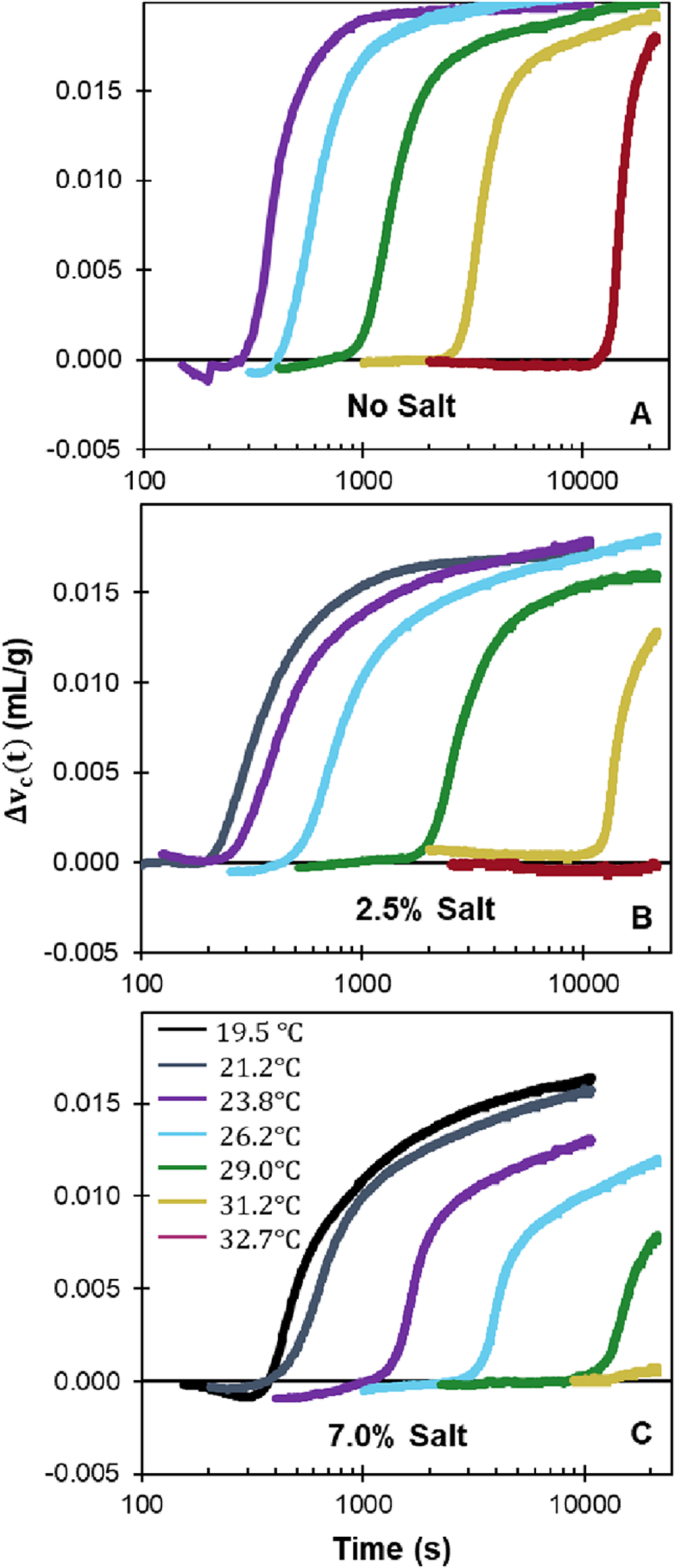
Time-evolution of specific volume change during crystallization at various temperatures. All samples have 20.0 wt % POCB, and salt weight percentages noted at the bottom of each figure.

As described previously,^6,7^ upon cooling to *T*_c_, two forms of volume change were observed. Within the first few minutes, the volume decreased due to thermal contraction as the samples cooled from 48 °C to *T*_c_. Over a longer period, there was a further volume decrease due to crystallization. This latter volume decrease occurred more quickly at lower crystallization temperatures, and hence at sufficiently low *T*_c_ values, crystallization started even before the thermal contraction was completed. Such fast crystallization is no longer isothermal and was not analyzed. The volume change associated with crystallization was obtained by subtracting the volume change due to thermal contraction from the total volume change.^6^Figure 4 shows examples of the specific volume change due to crystallization, Δ*v*_c_(*t*), for samples with 20 wt % POCB, without salt, with 2.5 wt % salt, and with 7.0 wt % salt. Other intermediate values of salt loadings are shown in Figure S3. As previously described,^6^ even though crystallization shrinks the volume of the mixture, Δ*v*_c_ is shown as a positive quantity.

Each crystallization trial follows a similar pattern of an initial latent period without significant crystallization, followed by rapid crystallization and an eventual plateau at some final value. The duration of the initial latent period grew with increasing salt content; compare for instance the time at which crystallization starts at 29 °C in Figure 4A (roughly 1000 s) vs 4B (roughly 2000 s). This trend points toward salt having an inhibiting effect on the cocrystallization kinetics of POCB hydrate. An analogous salt effect was described by Bianchi^27^ wherein the presence of salt inhibited the crystallization of poly(caproamide).

**Figure 5:**
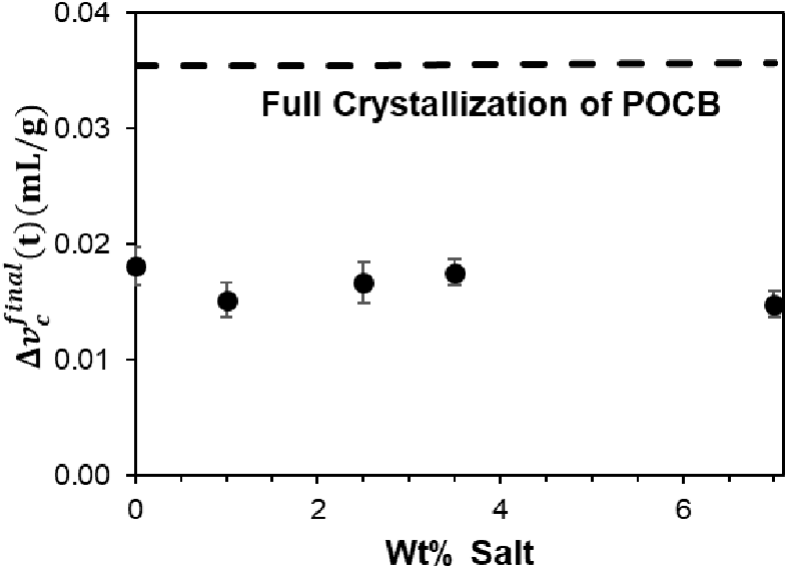
Average specific volume change at long crystallization times for samples with 20 wt % POCB. The dotted line indicates the theoretical maximum value predicted by eq 2.

As previously,^6^ we seek to quantify the crystallization behavior in terms of two parameters: the value of Δ*v*_c_ at long times , and the time needed to reach a specified value of Δ*v*_c_. To estimate , we plotted the value of Δ*v*_c_ at *t* = 10^4^*s* (selected arbitrarily) for each experiment. Figure S4 shows that this value increases with decreasing temperature *T*_c_, and then it approaches a plateau. This suggests that at sufficiently low temperature crystallization is complete by 10^4^ s.  was taken as the average of Δ*v*_c_ values from the six lowest temperatures available for each composition. Figure 5 shows that the  does not change any systematic way with salt content.

A theoretical maximum volume change,  (assuming all POCB is consumed to form hydrate), can be estimated as^6^
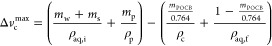
2where *ρ*_aq,i_, *ρ*_aq,f_, *ρ*_POCB_, and *ρ*_c_ are the densities of the aqueous phase before crystallization, the aqueous phase after crystallization, POCB, and the hydrate crystal respectively, and 0.764 is the weight fraction of POCB in the hydrate crystal. The term in the first brackets is the sum of the specific volume of the saltwater and liquid POCB, which are assumed to be completely immiscible. The term in the second brackets is the sum of the specific volumes of the hydrate crystal and the uncrystallized saltwater. The density of the crystal phase is *ρ*_c_ = 1.176 g/mL (based upon dimensions of the unit cell,^34^ and the density of POCB is *ρ*_POCB_ = 1.02 g/mL. The density of the saltwater phase is calculated for each composition based upon the model provided by Simion^35^ by assuming that all the salt is in the aqueous phase both before and after crystallization. Further all densities are assumed to be independent of temperature. The prediction of eq 2 (dashed line in Figure 5) is found to be nearly independent of salt content (in effect, *ρ*_aq,i_ and *ρ*_aq,f_ only weakly depends on salt content).

The predicted value is found to be nearly twice of the measured values of Δ*v*^final^ suggesting that only roughly 50% of the POCB actually crystallizes at any given salt content. This value of 50% crystallization is similar to that estimated from previous experiments in which the hydrate crystallized from a homogeneous liquid phase with an excess POCB.^6^ Yet, it should be noted that this conclusion of 50% crystallization depends on the estimated crystal phase density. Even a few percent error in the published values of unit cell dimensions change the *ρ*_c_ value, and hence the value of  significantly.^6^

**Figure 6:**
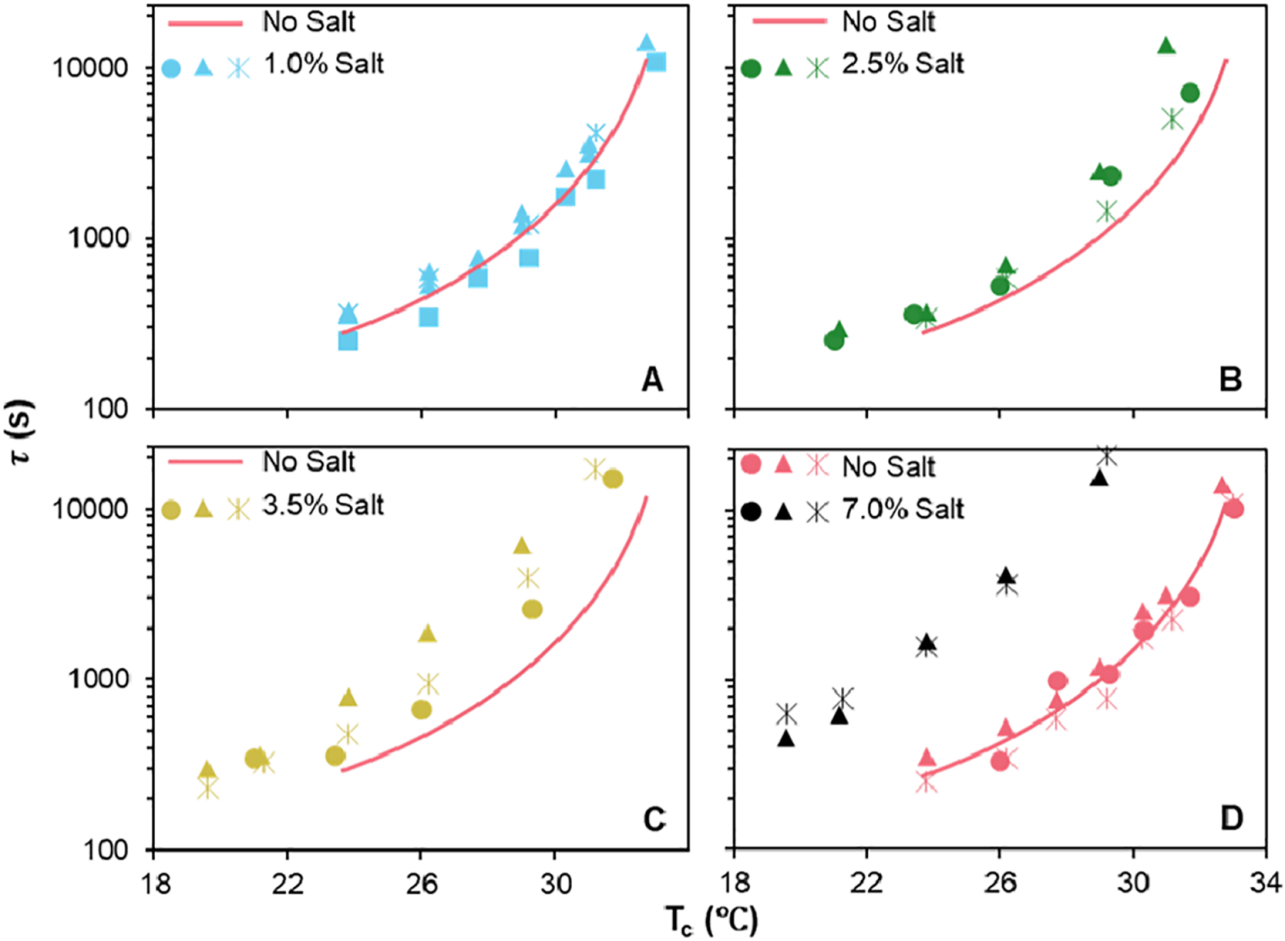
Time for Δ*v*_c_ = 0.005 mL/g at various crystallization temperatures. All samples have 20 wt % POCB, with varying salt contents. The pink data in D corresponds to the salt-free sample (reproduced with permission from ref (7) Copyright 2023 Elsevier-polymer) and the same pink line is repeated in A, B, and C as a guide. Different symbols in each graph represent distinct samples of the same composition.

Next, we turn to the effect of salt on the crystallization rate. As in our previous studies^6,7^ to quantify the kinetics, from each Δ*v*_c_(*t*) curve, we identified the time τ at which the volume change reached 0.005 mL/g (chosen arbitrarily), i.e., Δ*v*_c_(*t* = τ) = 0.005 *mL*/*g*. This time τ provides a single number to compare the kinetics data at all salt contents. Figure 6 shows τ versus crystallization temperature *T*_c_. The corresponding salt-free data are shown only in Figure 6D, but the solid pink line passing through the salt-free data is shown in Figures 6A-D for comparison. In general, increasing salt content increases τ, i.e., slows down crystallization, although the effect is modest below 3 wt % salt.

As mentioned at the beginning of this section, the salt-induced melting point depression reduces the undercooling *ΔT* = (*T*_m_*– T*_c_), and hence, it is expected to slow crystallization. To quantify the extent of this effect, we must therefore compare τ values at fixed undercooling, rather than at fixed *T*_c_. The well-established approach for doing so is the Hoffman–Lauritzen theory^36^ which relates the kinetics of crystallization to the free energy of secondary nucleation on the face of the growing crystal. It predicts crystallization rate as
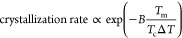
4Note that the quantity *T*_m_*/T*_c_ is close to 1 in our case, thus to a first approximation, the right-hand side depends on Δ*T* alone. Accordingly, eq 4 accounts for the changes in undercooling due to melting point depression; if that is the only factor by which salt affects crystallization kinetics, then one would expect data at all salt contents to collapse onto a single line. That would suggest that the only effect of salt is to slow down the secondary nucleation necessary for crystal growth.

**Figure 7:**
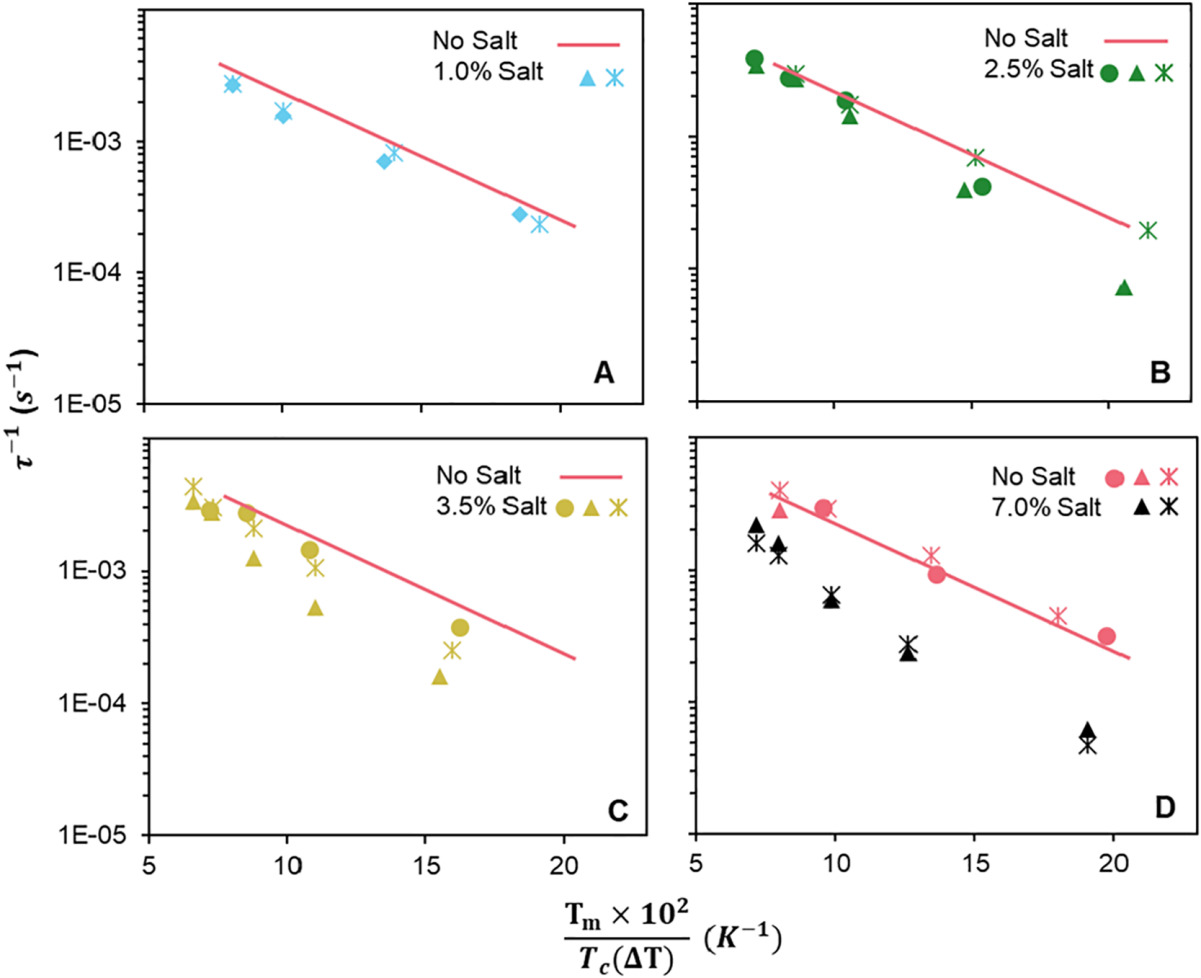
Time for Δ*v*_c_ = 0.005 mL/g for various salt contents in the form of a Hoffman–Lauritzen plot. Different symbols in each graph represent a distinct sample of the same composition. The pink data in D corresponds to the salt-free sample (reproduced with permission from ref (7) Copyright 2023 Elsevier-polymer). The pink straight line corresponds to eq 4 for the salt-free data and is reproduced in A-C as a reference.

Accordingly, with τ^–1^ as a measure of the crystallization rate, Figure 7 plots *ln*(τ^–1^) vs *T*_m_/*T*_m_Δ*T*. While the number of points for each sample are small, the data do appear approximately linear, consistent with eq 4, i.e., the Hoffman–Lauritzen model can represent the data at least approximately. A detailed discussion of the salt-free data was presented previously;^7^ here we only focus on the effects of salt. Even though data at the different salt contents do move closer together than in Figure 6, data for the various salt samples remain distinctly below the salt-free line. These results indicate that the salt effects on undercooling can account for only a part of the slowing of the crystallization kinetics. We emphasize that this last conclusion does not rely on the validity of the Hoffman–Lauritzen model. The effect of salt-induced melting point depression can be judged without guidance from Eq 4: Simply plotting the results of Figure 6 with Δ*T* on the *x*-axis shows that, even when compared at fixed undercooling, salt slows hydrate crystallization. Finally, the samples at 2.5 and 3.5% show a large degree of sample-to-sample variability with some behaving similar to the sample without salt, and some to the sample with 7 wt % salt. It is possible that the mechanism of crystallization changes across this concentration range.

Figure 7 also shows a distinct increase in the temperature-dependence of the crystallization kinetics with increasing salt content. For homopolymers, a decrease in slope of the crystallization kinetics vs *T*_m_/*T*_m_Δ*T* indicates a decrease in the surface energy at the crystal-melt interface as per the Hoffman–Lauritzen model.^36^ Accordingly, Figure 7 indicates that increasing salt concentration reduces the surface energy. In the current situation, however, hydrate crystallization occurs from a mixture of polymer, salt, and water. For a homopolymer crystallizing from a mixture, there is an additional contribution to the slope that comes from the concentration of the polymer.^37^ Although we keep the polymer loading fixed at 20 wt % in all samples, the compositions of the coexisting liquid phases may change with salt content and may in turn affect the temperature-dependence. Thus, it is difficult to conclusively pin down the reason underlying the increasing temperature-sensitivity of the crystallization kinetics with increasing salt content.

As in Banerjee et al.,^7^ we also considered an alternate method to quantify kinetics which is based on the induction time, i.e., the time up to which Δ*v*_c_ remains nearly zero (see short time behavior in Figure 5). This induction time, *t*_ind_, has been regarded as the time for primary nucleation^38^ and serves as another measure of crystallization kinetics. Analysis of data based on the induction time (see ESI) gives conclusions that are essentially identical to those based on analysis using the time scale τ.

## Summary and Conclusions

To summarize, inorganic salts are well known to induce liquid–liquid separation in aqueous solutions of many water-soluble polymers. This paper examines the effects of adding salt to mixtures of polyoxacyclobutane (POCB) and water. POCB has the rare ability to cocrystallize with water to form a hydrate, one of only two or three polymers that can do so. Further, above the melting temperature of the hydrate, mixtures of POCB and water can exist in liquid–liquid equilibrium (LLE). This combination of immiscibility in the liquid phase and cocrystallization at low temperature gives POCB–water mixtures a phase diagram that is distinct from all other polymer–solvent pairs. Here, we report on the effect of adding NaCl salt to the phase diagram of POCB–water mixtures and to the kinetics of hydrate crystallization.

The central results of this paper are as follows.

1. Even low loadings of salt (less than 0.1 wt %) can significantly expand the composition range within which LLE appears, i.e., there is a salting-out effect similar to other aqueous polymer solutions. The expansion of LLE, as judged by lowering of the cloud point temperature, is especially severe at low water content. This suggests that the reason why salt drives LLE is that by demixing of the polymer and water and partitioning into the aqueous phase, the salt can avoid association with the polymer.

2. Much larger salt loadings (∼10 wt %) reduce the melting temperature of the hydrate.

3. The hydrate is remarkably salt-tolerant and can coexist even with salt-saturated water. Accordingly, the lowest melting point of the hydrate (i.e., the greatest melting point depression of the hydrate) corresponds to a four-phase equilibrium between a POCB-rich liquid phase, a water-rich liquid phase, solid hydrate, and solid NaCl. The corresponding four-phase equilibrium temperature is 26 °C, just above room temperature.

4. Since salt greatly shrinks the homogeneous liquid region, in POCB–water–salt mixtures, hydrate crystallization starts from the LLE state at almost all compositions. Bulk crystallization experiments (conducted with continuous stirring to avoid layering of the two liquids) show that salt decreases the crystallization rate. The decrease in rate is modest for ∼1 wt % salt loading but approaches 10x at the highest loading (7% salt) examined. Some, but not all, of the slowing of hydrate crystallization can be explained by the melting point depression, i.e., the fact that at any given crystallization temperature, salt-containing samples are at lower undercooling.

To the best of our knowledge, the latter two items are the first report of any salt effect on the phase behavior or crystallization kinetics of a polymer hydrate cocrystal.

Overall, the effect of salt on the LLE behavior of POCB–water mixtures is similar to that of other aqueous polymer solutions. The effect of salt on the hydrate melting point is qualitatively similar to the melting point depression of any crystal equilibrating with a solute. Finally, the fact that salt slows the hydrate crystallization even when compared at fixed undercooling suggests that salt may have additional interfacial effects that slow crystallization beyond what may be expected from melting point depression alone.

## Experimental

### Materials

Polyoxacyclobutane was obtained from DuPont under the trade name Cerenol. The experiments used POCB with a number-average molecular weight of 650 g/mol, as reported by the manufacturer. The glassware used in the dilatometry experiments was prepared in a glass shop at the University of Pittsburgh. As even small salt impurities were found to significantly alter cloud-point results, Millipore water was used in all samples.

### Cloud Point Methods

POCB–water–salt mixtures (roughly 3 g total) were weighed into small glass vials. The vial lid was closed and sealed with a silicone sealant to prevent any water loss due to evaporation. The samples were immersed in a water bath with the temperature controlled by a hot plate. The bath temperature was gradually raised until sample cloudiness became visually apparent and then lowered to verify the disappearance of cloudiness. For these experiments, it was necessary for the entire vial to be immersed in a water bath. If any portion of the vial, e.g., the cap, was exposed to cooler air during the experiment, condensation of water on the cooler surface confounded the results.

### Melting Point Methods

POCB–water–salt mixtures were weighed into small glass vials and allowed to crystallize at room temperature over many days. A small quantity of the resulting hydrate–liquid mixture was then placed into a polystyrene well plate and flattened. This well plate was placed under a microscope in a temperature-controlled stage and heated in 2 °C increments starting from 22 °C. The temperature at which the hydrate crystals were no longer visible was taken as the melting point temperature of that sample.

### Crystallization Measurement Methods

POCB–water–salt mixtures (roughly 1 g in total) were weighed into round-bottom flasks. A magnetic stir bar was added to all samples. The samples were then covered with mineral oil, and the flasks were joined to a capillary stem and sealed with parafilm. The samples were heated to 48 °C to ensure all hydrate crystals were melted and then cooled rapidly in a water bath set to the desired crystallization temperature. All the graphs and the data analysis use the true temperature that was continuously read off a calibrated digital thermometer immersed in the water bath. Typically, the temperature during any single experiment varied no more than ±0.1 °C. The samples were stirred continuously during this process (see the next paragraph). The change in volume was measured by imaging the oil meniscus level in the capillary tube and then analyzing the images using Blender motion tracking to determine the meniscus position over time. The volume changes were then calculated and normalized by the sample weight. The procedure and data analysis are explained in detail in our previous study.^6^ The camera resolution allows the change in height of the oil meniscus to be measured with 0.02 mm accuracy. Combined with the cross-sectional area of the capillary, the corresponding volume change is around 0.0007 mL, whereas typical volume changes in each experiment are roughly 0.05 mL. We believe that the greatest variability in the experiments comes from the fact that mixing may not be perfectly reproducible in such small samples. For example, if a large drop of the mixture becomes entrained in the oil phase early during the experiment, it may crystallize earlier or later than the rest of the mixture.

Since samples are in liquid–liquid equilibrium prior to crystallization, the rate of mixing was found to have a significant effect on the kinetics of crystallization. As such, a rotor assembly was designed and implemented to ensure similar mixing in each sample. The rotor assembly consists of five rotors connected by intermeshed gears and is driven by a single motor that drives the center gear by an elastic band. The motor rotation speed was set to 140 rpm for all experiments.

## Data Availability

Experimental data, including the evolution of volumes with time, and spreadsheets of the data used to plot Figures 2, 3, 4, and 6 will be available on request.

## Supporting Information Available

Additional experimental data analysis of crystallization kinetics including figures and alternate method of quantifying crystallization kinetics. Also, microscopic images of hydrate melting point, salt crystals (PDF)

## References

[ref1] Tadokoro H.; Takahashi Y.; Chatani Y.; Kakida H. Structural studies of polyethers, [—(CH2)m—O—]n.V. Polyoxacyclobutane. Makromol. Chem. 1967, 109, 96–111. 10.1002/macp.1967.021090110.

[ref2] Banerjee J.; Koronaios P.; Morganstein B.; Geib S. J.; Enick R. M.; Keith J. A.; Beckman E. J.; Velankar S. S. Liquids That Freeze When Mixed: Cocrystallization and Liquid–Liquid Equilibrium in Polyoxacyclobutane–Water Mixtures. Macromolecules 2018, 51, 3176–3183. 10.1021/acs.macromol.8b00239.

[ref3] Chatani Y.; Kobatake T.; Tadokoro H. Structural studies of poly(ethylenimine). 3. Structural characterization of anhydrous and hydrous states and crystal structure of the hemihydrate. Macromolecules 1983, 16 (2), 199–204. 10.1021/ma00236a009.

[ref4] Benkhira A.; Franta E.; Francois J. Polydioxolane in aqueous solutions. 1. Phase diagram. Macromolecules 1992, 25 (21), 5697–5704. 10.1021/ma00047a022.

[ref5] Guenet J.-M. Polymer-Solvent Molecular Compounds; Elsevier, 2010

[ref6] Barker E.; Banerjee S.; Meyer T. Y.; Velankar S. Liquids that Freeze when Mixed: Homogeneous Cocrystallization Kinetics of Polyoxacyclobutane–Water Hydrate. ACS Appl. Polym. Mater. 2022, 4, 703–713. 10.1021/acsapm.1c01626.

[ref7] Banerjee S.; Gresh-Sill M.; Barker E. F.; Meyer T. Y.; Velankar S. S. Polymer co-crystallization from LLE: Crystallization kinetics of POCB hydrate from two-phase mixtures of POCB and water. Polymer 2023, 282, 126087 10.1016/j.polymer.2023.126087.

[ref8] Chido M. T.; Koronaios P.; Saravanan K.; Adams A. P.; Geib S. J.; Zhu Q.; Sunkara H. B.; Velankar S. S.; Enick R. M.; Keith J. A. Oligomer hydrate crystallization improves carbon nanotube memory. Chem. Mater. 2018, 30, 3813–3818. 10.1021/acs.chemmater.8b00964.

[ref9] Badeau B. A.; DeForest C. A. Programming stimuli-responsive behavior into biomaterials. Annu. Rev. Biomed. Eng. 2019, 21 (1), 241–265. 10.1146/annurev-bioeng-060418-052324. 30857392

[ref10] Municoy S.; Álvarez Echazú M. I.; Antezana P. E.; Galdopórpora J. M.; Olivetti C.; Mebert A. M.; Foglia M. L.; Tuttolomondo M. V.; Alvarez G. S.; Hardy J. G. Stimuli-responsive materials for tissue engineering and drug delivery. Int. J. Mol. Sci. 2020, 21, 4724 10.3390/ijms21134724. 32630690 PMC7369929

[ref11] Babu P.; Nambiar A.; He T.; Karimi I. A.; Lee J. D.; Englezos P.; Linga P. A Review of Clathrate Hydrate Based Desalination To Strengthen Energy–Water Nexus. ACS Sustainable Chem. Eng. 2018, 6, 8093–8107. 10.1021/acssuschemeng.8b01616.

[ref12] Freier E.; Wolf S.; Gerwert K. Proton transfer via a transient linear water-molecule chain in a membrane protein. Proc. Natl. Acad. Sci. U. S. A. 2011, 108 (28), 11435–11439. 10.1073/pnas.1104735108. 21709261 PMC3136293

[ref13] Gadjourova Z.; Andreev Y. G.; Tunstall D. P.; Bruce P. G. Ionic conductivity in crystalline polymer electrolytes. Nature 2001, 412, 520–523. 10.1038/35087538. 11484048

[ref14] Wei M.; Gao Y.; Li X.; Serpe M. J. Stimuli-responsive polymers and their applications. Polym. Chem. 2017, 8, 127–143. 10.1039/C6PY01585A.

[ref15] Albertsson P.-Å. Aqueous Polymer-Phase Systems. In Partition of Cell Particles and Macromolecules; Wiley: New York, 1986; 8–39

[ref16] Lee H.-N.; Rosen B. M.; Fenyvesi G.; Sunkara H. B. UCST and LCST phase behavior of poly(trimethylene ether) glycol in water. J. Polym. Sci. A Polym. Chem. 2012, 50, 4311–4315. 10.1002/pola.26242.

[ref17] Liu Y.; Wang Y.; Cai W. Salting Effect of Sodium Hydroxide and Sodium Formate on the Liquid–Liquid Equilibrium of Polyoxymethylene Dimethyl Ethers in Aqueous Solution. Journal Of Chemical & Engineering Data 2019, 64 (6), 2578–2592. 10.1021/acs.jced.9b00081.

[ref18] Ananthapadmanabhan K. P.; Goddard E. D. Aqueous biphase formation in polyethylene oxide-inorganic salt systems. Langmuir 1987, 3 (1), 25–31. 10.1021/la00073a005.

[ref19] Thormann E. On understanding of the Hofmeister effect: how addition of salt alters the stability of temperature responsive polymers in aqueous solutions. RSC Adv. 2012, 2, 8297 10.1039/c2ra20164j.

[ref20] Zafarani-Moattar M. T.; Sadeghi R. Measurement and correlation of liquid–liquid equilibria of the aqueous two-phase system polyvinylpyrrolidone–sodium dihydrogen phosphate. Fluid Phase Equilib. 2002, 203 (1), 177–191. 10.1016/S0378-3812(02)00179-6.

[ref21] Zhang Y.; Furyk S.; Sagle L. B.; Cho Y.; Bergbreiter D. E.; Cremer P. S. Effects of Hofmeister Anions on the LCST of PNIPAM as a Function of Molecular Weight. J. Phys. Chem. C 2007, 111, 8916–8924. 10.1021/jp0690603. PMC2553222 18820735

[ref22] Herbst J.; Pott R. W. M. The Effect of Temperature on Different Aqueous Two-Phase Diagrams of Polyethylene Glycol (PEG 6000, PEG 8000, and PEG 10000) + Potassium Sodium Tartrate + Water. Journal Of Chemical & Engineering Data 2019, 64 (7), 3036–3043. 10.1021/acs.jced.9b00133.

[ref23] Sosa F. H. B.; Farias F. O.; Igarashi-Mafra L.; Mafra M. R. Measurement and correlation of aqueous two-phase systems of polyvinylpyrrolidone (PVP) and manganese sulfate: Effects of molecular weight and temperature. Fluid Phase Equilib. 2018, 472, 204–211. 10.1016/j.fluid.2018.05.021.

[ref24] Jana S.; Uchman M. Poly(2-oxazoline)-based stimulus-responsive (Co)polymers: An overview of their design, solution properties, surface-chemistries and applications. Prog. Polym. Sci. 2020, 106, 101252 10.1016/j.progpolymsci.2020.101252.

[ref25] Zaslavsky B. Y.; Bagirov T. O.; Borovskaya A. A.; Gasanova G. Z.; Gulaeva N. D.; Levin V. Y.; Masimov A. A.; Mahmudov A. U.; Mestechkina N. M.; Miheeva L. M.; Osipov N. N.; Rogozhin S. V. Aqueous biphasic systems formed by nonionic polymers I. Effects of inorganic salts on phase separation. Colloid. Polymer Sci. 1986, 264, 1066–1071. 10.1007/BF01410324.

[ref26] Hyde A. M.; Zultanski S. L.; Waldman J. H.; Zhong Y.-L.; Shevlin M.; Peng F. General Principles and Strategies for Salting-Out Informed by the Hofmeister Series. Org. Process Res. Dev. 2017, 21, 1355–1370. 10.1021/acs.oprd.7b00197.

[ref27] Bianchi E.; Ciferri A.; Tealdi A.; Torre R.; Valenti B. Bulk Properties of Synthetic Polymer-Inorganic Salt Systems. II. Crystallization Kinetics of Salted Poly(caproamide). Macromolecules 1974, 7, 495–500. 10.1021/ma60040a017.

[ref28] Valenti B.; Bianchi E.; Tealdi A.; Russo S.; Ciferri A. Bulk Properties of Synthetic Polymer-Inorganic Salt Systems. IV. Role of the Polymeric Substrate. Macromolecules 1976, 9, 117–122. 10.1021/ma60049a023.

[ref29] Hashifudin A.; Sim L. H.; Chan C. H.; Ramli H. Phase behaviour and morphology of composite comprising of poly(ethylene oxide), polyacrylate and lithium perchlorate. Compos. Interfaces 2014, 21, 797–805. 10.1080/15685543.2014.960321.

[ref30] Dendy Sloan E.; Koh C. A. Chapter 4: Effect of Thermodynamic Inhibitors on Hydrate Formation. In Clathrate Hydrates of Natural Gases; CRC Press: Boca Raton, FL, 2008; pp 229–236

[ref31] Florin E.; Kjellander R.; Eriksson J. C. Salt effects on the cloud point of the poly(ethylene oxide)+ water system. J. Chem. Soc., Faraday Trans. 1 1984, 80 (11), 2889 10.1039/f19848002889.

[ref32] Huddleston J. G.; Willauer H. D.; Griffin S. T.; Rogers R. D. Aqueous Polymeric Solutions as Environmentally Benign Liquid/Liquid Extraction Media. Ind. Eng. Chem. Res. 1999, 38, 2523–2539. 10.1021/ie980505m.

[ref33] Sadeghi R.; Jahani F. Salting-In and Salting-Out of Water-Soluble Polymers in Aqueous Salt Solutions. J. Phys. Chem. B 2012, 116 (17), 5234–5241. 10.1021/jp300665b. 22486327

[ref34] Kakida H.; Makino D.; Chatani Y.; Kobayashi M.; Tadokoro H. Strutural Studies of Polyethers [-(CH2)mO-]n. VIII. Polyoxacyclobutane Hydrate (Modification I). Macromolecules 1970, 3, 569–578.

[ref35] Simion A. I.; Grigoras C.-G.; Rosu A.-M.; Gavrila L. Mathematical Modelling of Denisty and Viscosity of NACL Aqueous Solutions. Journal Of Agroalimentary Porcesses and Technologies 2014, 21, 41–52.

[ref36] Mandelkern L. Crystallization of Polymers: Kinetics & Mechanism, 2nd ed.; Cambridge University Press: New York, USA, 2004; 2, Chapter 9

[ref37] Mandelkern L. Crystallization of Polymers: Kinetics & Mechanism, 2nd ed.; Cambridge University Press: New York, USA, 2004; 2, Chapter 11

[ref38] Iwamatsu M. Direct numerical simulation of homogeneous nucleation and growth in a phase-field model using cell dynamics method. J. Chem. Phys. 2008, 128 (8), 084504 10.1063/1.2883652. 18315058

