# Supplementary material for: Salt Effects on the Phase Behavior and Cocrystallization Kinetics of POCB–Water Mixtures
Source: Langmuir. 2024 Feb 2;40(6):2862–71. doi: 10.1021/acs.langmuir.3c02428 (PMC10867884; doi:10.1021/acs.langmuir.3c02428)
Supplement: Supplementary file 1 — la3c02428_si_001.pdf [file la3c02428_si_001.pdf]

Supplemental Information

Salt effects on the phase behavior and co-crystallization kinetics of POCB-water mixtures

Michael Gresh-Sill[1], Sudesna Banerjee[1], Tara Y. Meyer[2] Sachin S. Velankar[1,3]

[1]Department of Chemical Engineering, [2]Department of Chemistry, [3]Department of Mechanical Engineering, University of Pittsburgh, Pittsburgh, PA, United States.

Quantifying salt partitioning into the POCB-rich phase

To measure the salt partitioning in the POCB-rich phase, a sample of the POCB was poured onto a layer of water saturated with salt at 40C. After one day of equilibration, roughly 1 g the POCB-rich phase was carefully removed excluding any contamination with the saltwater. This was then equilibrated with 20 times the mass of water at room temperature to allow the POCB to form hydrate. The salt content in the water was then measured by solution conductivity measurements and found to be 5 ppm. On the assumption that the salt entirely partitions into the large excess of water, the salt content of the POCB-rich phase was calculated to be 20 times higher, i.e. 100 ppm or 0.01%. Since the original POCB-rich phase was in equilibrium with salt-saturated water, this value of 0.01% may be regarded as an upper limit to the solubility of salt in the POCB-rich phase.

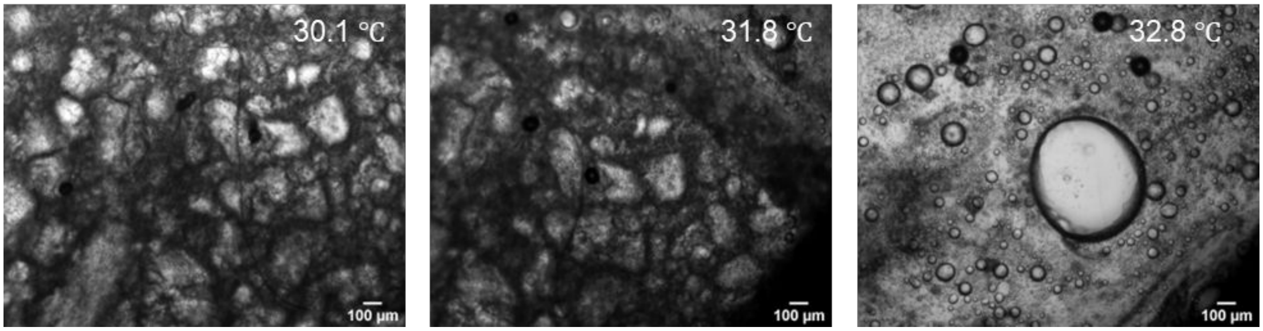

Figure S1: Example of hydrate melting point measurements using optical microscopy. Images show a single sample of composition 60 wt% POCB and 6% salt at varying temperatures. Hydrate melts between the second and the third image.

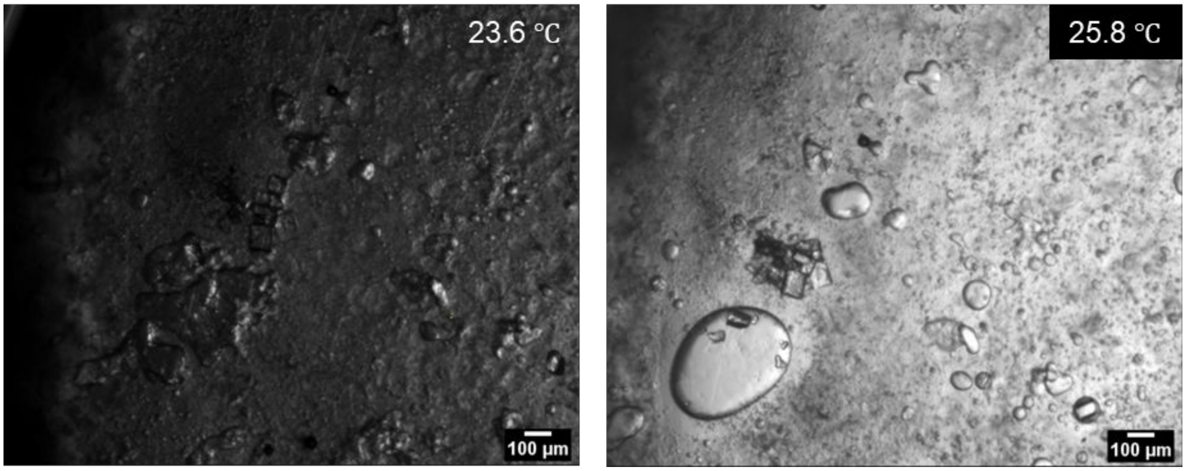

Figure S2: Example of salt crystals present in an 80% POCB 6% hydrate melting point sample. The salt crystals are the distinctly cube shaped particles, which remain present even after the hydrate has melted.

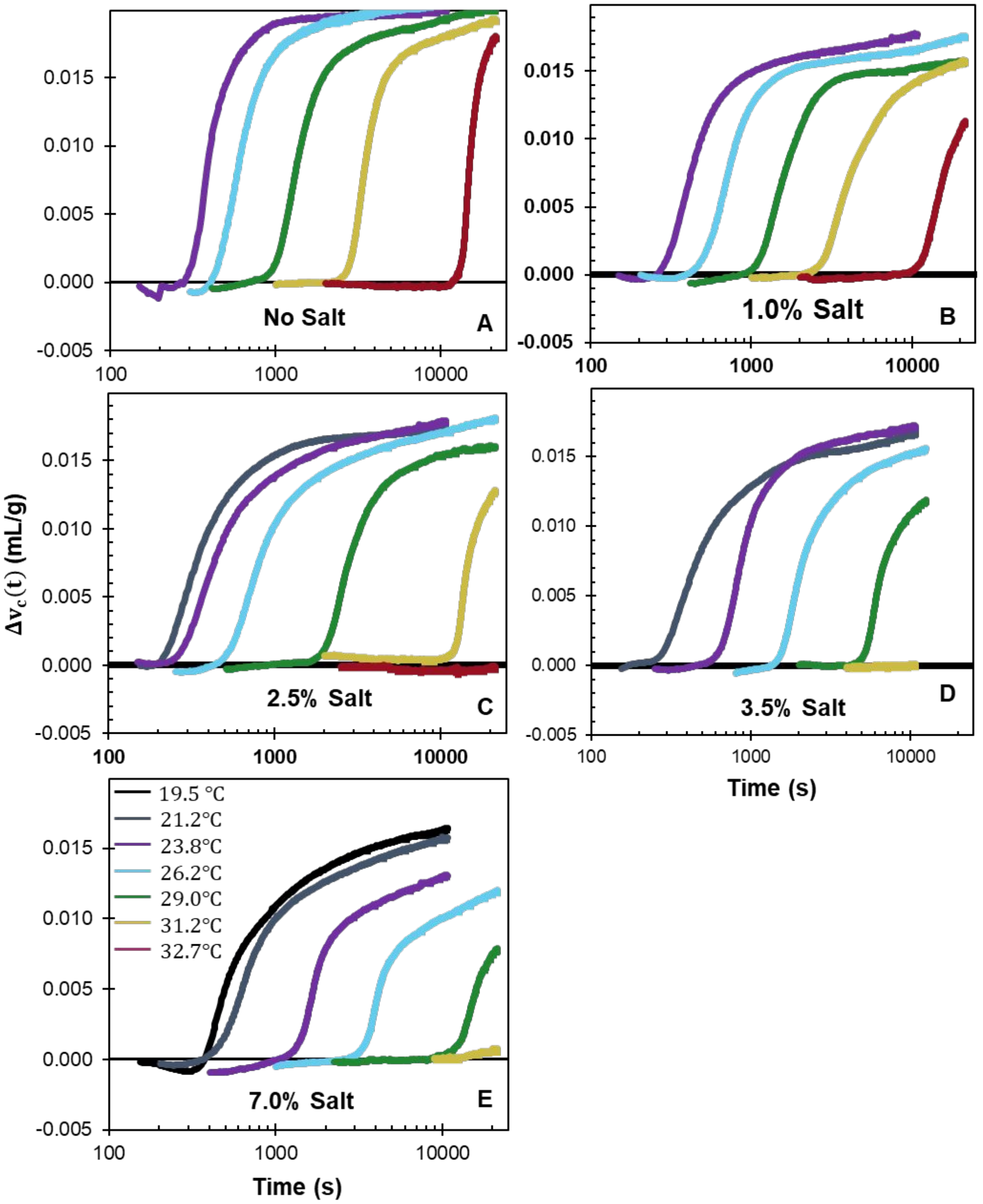

Figure S3: Bulk kinetic data for various salt loadings noted at the bottom of each graph.

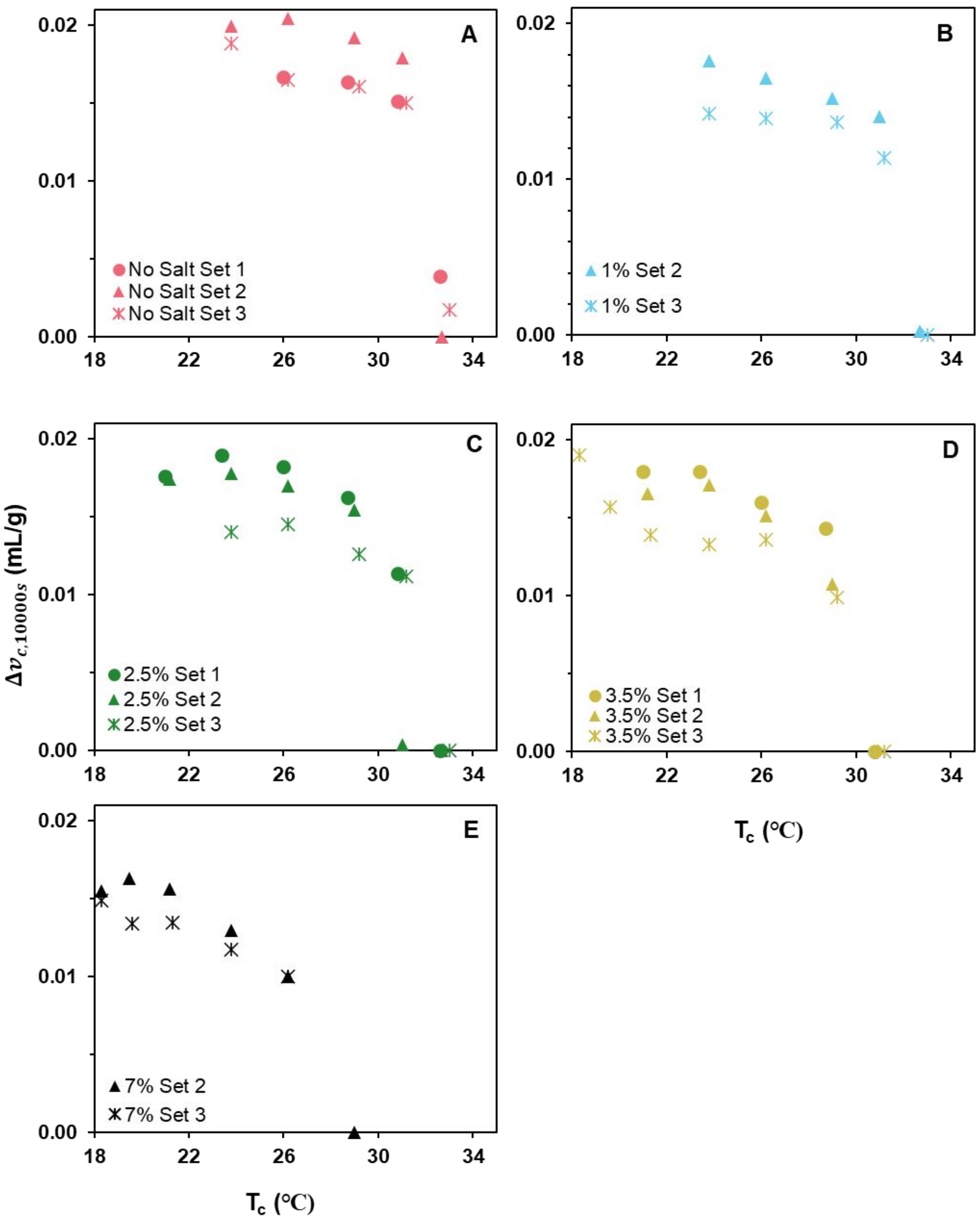

Figure S4: $\Delta v_c(t = 10^4 s)$ vs T_c. Multiple samples were prepared at each salt content, and crystallized at different values of T_c. Each individual sample is shown by a different symbol.

Alternate method to quantify kinetics

As in Banerjee et al [7], we also considered a second way to quantify kinetics. Figure 5 shows that Δv_c remains nearly zero for a certain period, often called the induction time. This induction time, t_{ind} has been regarded as the time for primary nucleation [37] and serves as another measure of crystallization kinetics.

To obtain t_{ind}, the $\Delta v_c(t)$ were fitted to the Avrami equation [38] :

$$\Delta v_c = \Delta v_c^{final} (1 - \exp(-k(t - t_{ind})^n) \quad \text{(S1)}$$

where n is the Avrami exponent, k is the Avrami coefficient, Δv_c^{final} is the volume change at long times. We acknowledge that the central assumptions of Avrami equation are not valid here since the samples are in LLE and under constant stirring. Accordingly, no physical significance can be attributed to n; we use Eq. S1 solely as a means of fitting the $\Delta v_c(t)$ data to estimate t_{ind} consistently across all samples. The data were fitted in a linearized form using Δv_c^{final}, k, t_{ind}, and n as fitting parameters. The resulting fits are shown in linearized form, in Figure S5 for each salt content. The values of Avrami exponent (n) vary across a wide range from 1.0 to 3.8 and increase with T_c (Figure S6).

These two measures of crystallization kinetics, τ and t_{ind} may now be compared. Figure S7 shows that the ratio τ/t_{ind} is typically about 1.5-2, albeit with significant variability, but there is no obvious trend with crystallization temperature. Thus, both measures of crystallization kinetics show roughly the same temperature-dependence, i.e. either can be used to judge the dependence of hydrate crystallization kinetics on salt content, and identical conclusions are reached in either case.

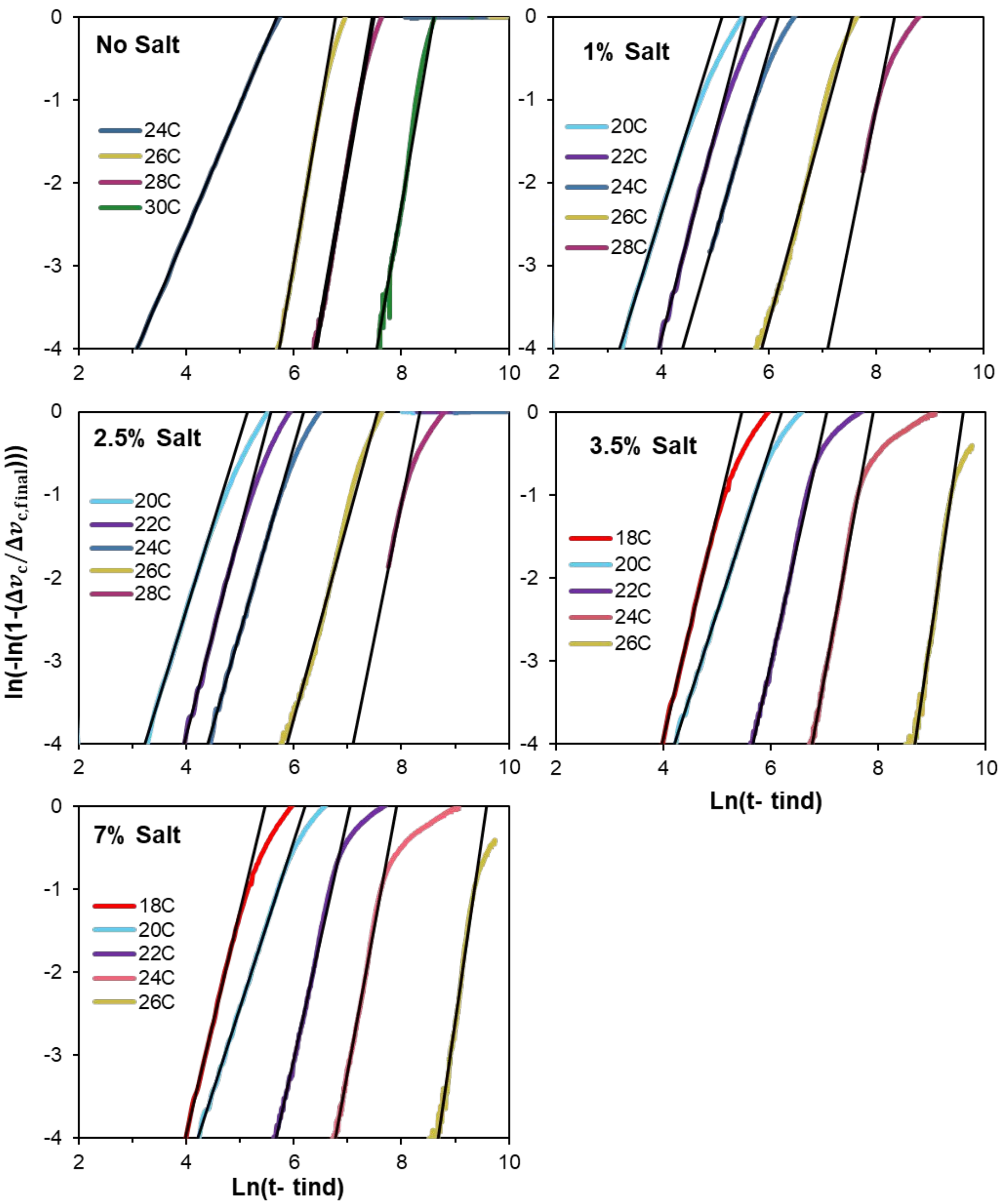

Figure S5 Specific volume change due to crystallization with Avrami equation fits overlayed. Same data as Figure S2 are used here.

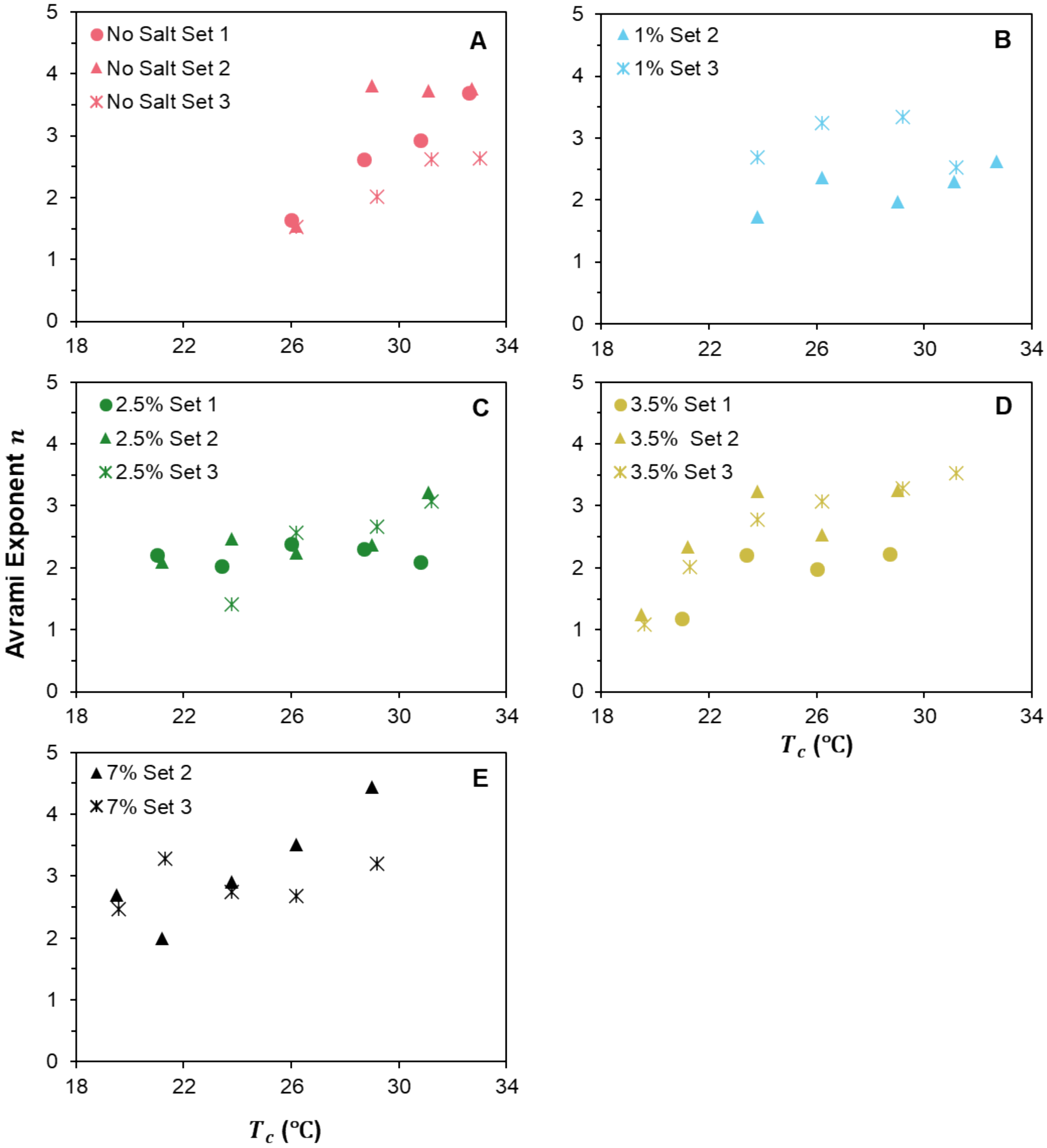

Figure S6 Avrami exponent n vs T_c

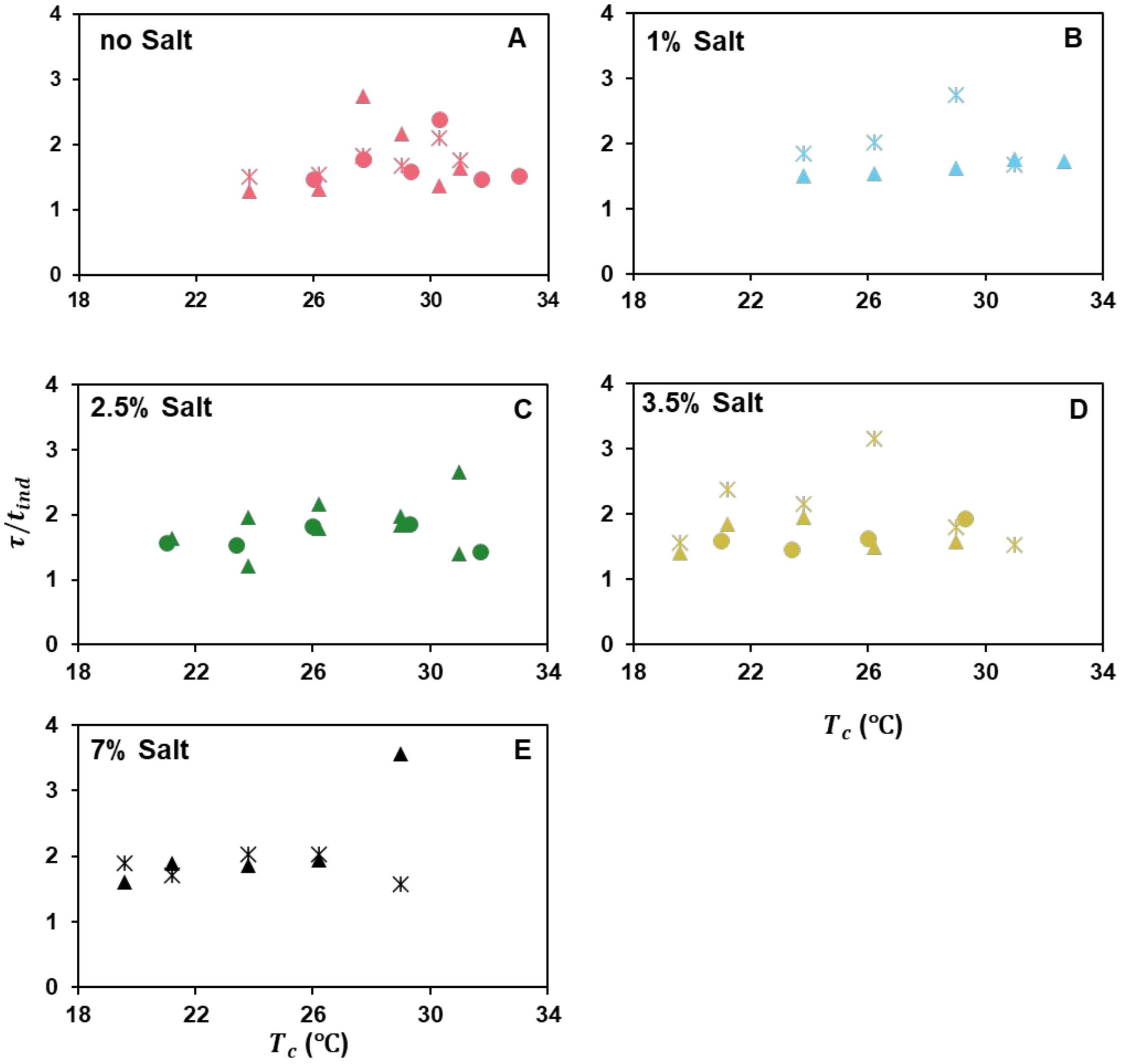

Figure S7 τ/t_{ind} versus crystallization temperature for samples at various salt contents
